# Biventricular Takotsubo Cardiomyopathy as an Unusual Presentation of SARS-CoV-2 mRNA Vaccine-Associated Multisystemic Inflammatory Syndrome

**DOI:** 10.7759/cureus.41365

**Published:** 2023-07-04

**Authors:** Kevin J. Arellano-Arteaga, Nikolai Emmanuel Bayro Jablonski, Elvira Miramontes Luna, Martín Bedolla-Barajas, Luz Gardenia Coronel Castañeda

**Affiliations:** 1 Department of Clinical Medicine/Internal Medicine, University Center for Health Science, University of Guadalajara, Guadalajara, MEX; 2 Department of Internal Medicine, Nuevo Hospital Civil de Guadalajara Dr. Juan I. Menchaca, Guadalajara, MEX; 3 Department of Allergy and Clinical Immunology, Nuevo Hospital Civil de Guadalajara Dr. Juan I. Menchaca, Guadalajara, MEX

**Keywords:** takotsubo cardiomyopathy, covid-19 vaccines, covid-19 related, biventricular takotsubo cardiomyopathy, multisystemic inflammatory syndrome

## Abstract

Biventricular takotsubo cardiomyopathy (BiTCM) is a rare entity, seldom reported. We describe a case of a female presenting with multisystemic inflammatory syndrome associated with the administration of a vaccine against coronavirus disease 2019 (COVID-19). In this particular case, the patient experienced the onset of symptoms shortly after receiving a COVID-19 vaccine. Early recognition of biventricular takotsubo cardiomyopathy and timely initiation of appropriate treatment are crucial. Prompt management includes stabilizing the patient's hemodynamic status, alleviating symptoms, and addressing any underlying causes, such as inflammation or immune-related responses. Close clinical surveillance is necessary to monitor the patient's cardiac function, assess response to treatment, and prevent potential complications.

## Introduction

The coronavirus disease 2019 (COVID-19) pandemic, caused by the severe acute respiratory syndrome coronavirus 2 (SARS-CoV-2), has affected millions of people worldwide. Since the beginning of the pandemic, multiple strategies have been implemented to control the spread of the virus, including the rapid production and distribution of safe and effective vaccines. As large-scale vaccination programs were implemented, reports of rare adverse events associated with some SARS-CoV-2 vaccines emerged [1]. One of these adverse events is the messenger RNA (mRNA) vaccine-associated multisystemic inflammatory syndrome (MIS-AV). This syndrome is characterized by systemic inflammation and multisystem involvement and can present with cardiovascular, gastrointestinal, cutaneous, and respiratory manifestations, among others [2,3].

In this report, we describe an unusual clinical case of a 59-year-old female who developed biventricular takotsubo cardiomyopathy (BiTCM) as an atypical manifestation of MIS-AV associated with the SARS-CoV-2 vaccine. Takotsubo cardiomyopathy (TCM) is an acquired disease characterized by reversible ventricular dysfunction, typically triggered by acute physical or emotional stress. However, its association with SARS-CoV-2 mRNA vaccines and biventricular presentation are infrequently reported and poorly described in the medical literature [4]. It has been described in a few cases associated with COVID-19 [5]. As far as we know, this is the only case of takotsubo cardiomyopathy with biventricular involvement associated with the SARS-CoV-2 vaccine. The aim of this case report is to highlight the importance of considering takotsubo cardiomyopathy as a possible complication of MIS-AV related to SARS-CoV-2. Additionally, we emphasize the need for continuous and comprehensive surveillance of vaccine-associated adverse events to improve the understanding of their pathophysiology and optimize the clinical care of affected patients.

This case report underscores the importance of multidisciplinary collaboration among internists, cardiologists, immunologists, and infectious disease specialists to appropriately address and treat these potential complications. As vaccination programs continue to be implemented, it is crucial to maintain close monitoring of vaccine-associated adverse events and share clinical experiences to advance knowledge and ensure the safety of SARS-CoV-2 vaccines.

## Case presentation

A 59-year-old female was referred to our institution for back pain and fever. She has a past medical history of stage IV non-small cell lung carcinoma with bone and brain metastases diagnosed in 2019 in remission, treated with afatinib and gamma Knife stereotactic radiosurgery for the brain lesions, and currently on osimertinib since 2021. Furthermore, she was diagnosed with idiopathic CD4+ lymphopenia in 2012 and chronic spontaneous angioedema in 2019 and was diagnosed with infectious mononucleosis in 2017.

Relevant personal history before the application of the second dose of the mRNA BNT162b2 SARS-CoV-2 vaccine 96 hours prior to hospital admission

Two days before admission, the patient started with a sharp, stinging lower back pain localized between L3 and L4, radiating to the ribs, without a recognizable temporal pattern, which worsened with physical activity. In addition, the patient reported a fever quantified in >38.5°C (101.3° F), controlled with acetaminophen. The following day, arthralgias and myalgias appeared, which led the patient to seek medical attention with an oncologist who started symptomatic treatment and referred the patient to the emergency department at our institution. Upon arrival, the patient presented with the described symptoms, as well as worsening back pain, fatigue, nausea, and vomiting. The Internal Medicine Department was consulted for evaluation.

Physical examination revealed a dry mouth, an erythematous oropharynx without discernible exudates, a tense right tympanic membrane on otoscopy, and palpable posterior cervical adenopathy <5 mm in diameter. In addition, the patient presented with abdominal pain on deep palpation without signs of peritoneal irritation or organomegaly. The rest of the examination, including the heart, was normal. On admission, a complete blood count (CBC) reported mild macrocytic, normochromic anemia, moderate hypovolemic hypotonic hyponatremia, and grade III hypocalcemia. Additionally, acute phase reactants were elevated, and procalcitonin was negative. Liver function tests reported a mild increase in transaminase levels, with normal bilirubin levels and mildly increased alkaline phosphatase (ALP) and lactate dehydrogenase (LDH) (Table 1). An electrocardiogram showed a regular rhythm with a prolonged corrected QT (QT: 490 msec), as well as T-wave inversions in V1-V4.

**Table 1:** Relevant laboratory test results Hb/cell: hemoglobin per cell, BUN: blood urea nitrogen, mg/dL: milligrams per deciliter, mL/minute/1.73 m^2^: milliliters per minute per 1.73 meters squared, mmol/L: millimoles per liter, g/dL: grams per deciliter, μm^3^: cubic micrometers, mm^3^: cubic millimeters, fL: femtoliters, ng/mL: nanograms per milliliter, pg/mL: picograms per milliliter, IL-6: interleukin-6, NT-proBNP: N-terminal pro-brain natriuretic peptide, SARS-CoV-2: severe acute respiratory syndrome coronavirus 2, PCR: polymerase chain reaction

Marker	Laboratory values	Reference range
Complete blood count		
Hemoglobin	10.8 g/dL	12.1-15.1 g/dL
Median corpuscular volume	97 μm^3^	80-100 μm^3^
Mean corpuscular hemoglobin	33 g/dL	27-34 g/dL
Platelet count	208,000 mm^3^	150,000-400,000 mm^3^
White blood cell count	7.6 × 10^9^/L	4.5-11 × 10^9^/L
Absolute neutrophil count	4.7 × 10^9^/L	2.5-7 × 10^9^/L
Absolute lymphocyte count	1.77 × 10^9^/L	0.8-5 × 10^9^/L
Complete metabolic panel		
Serum creatinine	0.62 mg/dL	0.7-1.3 mg/dL
BUN	13.07 mg/dL	7-20 mg/dL
Sodium	129 mmol/L	135-145 mmol/L
Potassium	3.7 mmol/L	3.7-5.1 mmol/L
Calcium	6.9 mmol/L	8.5-10.2 mmol/L
Chloride	91 mmol/L	96-106 mmol/L
Cardiac biomarkers		
NT-proBNP	>35,000 pg/mL	<125 pg/mL
Troponin I	0.19 ng/mL	<0.10 ng/mL
D-dimer	6,070 ng/mL	<500 ng/mL
Acute phase reactants		
C-reactive protein	82 mg/mL	<0.3 mg/dL
Erythrocyte sedimentation rate	30 mm/hour	0-20 mm/hour
Ferritin	280 ng/mL	24-307 ng/mL
IL-6	142 pg/mL	<7 pg/mL
Procalcitonin	0.05 ng/mL	<0.25 ng/mL
COVID-19 test		
SARS-CoV-2 antigen test	Negative	Negative
SARS-CoV-2 PCR test	Non-reactive	Non-reactive
Anti-SPIKE antibodies	Positive	N/A
Anti-SARS-CoV-2 antibodies (IgG)	Positive	Negative
Anti-SARS-CoV-2 antibodies (IgM)	Negative	Negative
Other laboratory tests		
Albumin	3.4 g/dL	3.4-5.4 g/dL
Alkaline phosphatase	145 U/L	44-147 U/L
Lactate dehydrogenase	204 U/L	33-105 U/L
Aspartate aminotransferase	69 U/L	8-33 U/L
Alanine aminotransferase	46 U/L	4-36 U/L

Differential diagnosis and workup

The patient was admitted to our institution for a workup of back pain with red flags (age, fever, and a history of cancer) and abdominal pain without peritoneal irritation in the context of an immunocompromised patient. A lumbar magnetic resonance imaging (MRI) with contrast was performed, showing no new changes in the vertebral metastatic activity, and a chest computed tomography (CT) scan showed minimal bilateral pleural effusion. A SARS-CoV-2 rapid antigen test and RT-PCR were negative, as well as a direct Coombs test and IgM for cytomegalovirus (CMV) and Epstein-Barr virus (EBV). An IgG anti-SARS-CoV-2 antibody and an anti-SPIKE SARS-CoV-2 antibody assay were positive. In addition, blood cultures were drawn. The patient was started on symptomatic treatment and kept on clinical surveillance. In the absence of clinical improvement, despite ruling out infectious diseases in the context of a systemic inflammatory response syndrome, treatment with intravenous corticosteroids was initiated. This treatment would help alleviate both the nonspecific inflammatory process and the associated pain. Furthermore, pain control measures were implemented using morphine and paracetamol to manage fever.

Day 3 of admission presented dyspnea, hypoxemia (SatO2: 86%), lower extremity edema, and ascites. Considering the diagnosis of acute heart failure, an echocardiogram was performed, reporting normal systolic and diastolic function, mild pulmonary hypertension, mild functional tricuspid regurgitation, and mild posterior pericardial effusion. Additionally, the patient presented with an NT-proBNP level greater than 35,000 and a slightly elevated troponin I measurement of 0.19 ng/mL. An elevated D-dimer led to the decision to perform a CT angiography, which revealed a worsening bilateral pleural effusion, with no evidence of pulmonary embolism. A diagnostic and therapeutic thoracocentesis revealed mononuclear transudate. The blood cultures came back negative. As a part of the clinical workup, antinuclear antibodies were performed, which were negative.

A clinical worsening of the edema 36 hours (day 5 of hospitalization) later prompted a clinical re-evaluation. A second electrocardiogram showed the persistence of the T-wave inversions in V3-V6, alongside a right bundle branch hemiblock. A strain transthoracic echocardiogram revealed findings consistent with myocarditis, reporting severe right and left ventricular systolic dysfunction without dilation, apical segmental akinesia, a now moderate pulmonary hypertension, a moderate biauricular dilation, an interauricular septal aneurysm type 1R, and mild functional mitral and tricuspid regurgitation, suspecting COVID-19 vaccine-related myocarditis. The patient was transferred to the intensive care unit, and heart failure treatment was instituted. Throughout her admission, electrocardiogram and troponin determinations did not show signs of acute myocardial infarction.

**Figure 1:**
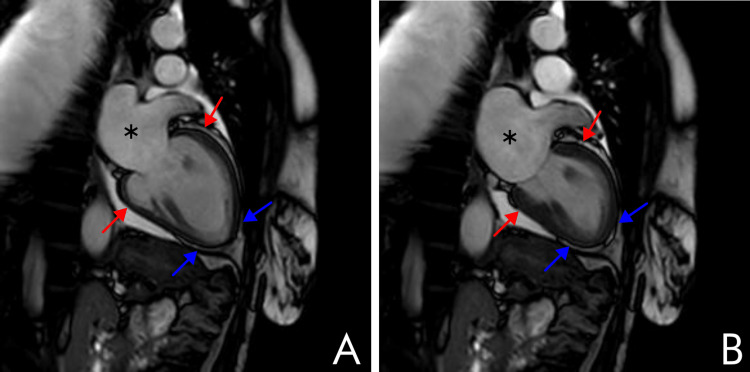
Cardiac MRI showing biventricular takotsubo cardiomyopathy T2 sequence of a cardiac MRI in sagittal view showing two moments of the cardiac cycle, diastole (A) and systole (B), respectively. The study reveals a hyperkinetic apical third (red arrows) with an akinetic basal third (blue arrows). Additionally, there is severe left atrial dilation (black asterisks), alongside a mild pericardial effusion in lateral and inferior walls. No late enhancement was reported. These findings suggest biventricular takotsubo cardiomyopathy and effectively rule out inflammatory myocarditis. MRI: magnetic resonance imaging

On day 7 of hospitalization, the peripheral edema decreased. A control echocardiogram reported improved biventricular function, with persistent severe left atrial dilation, and a significant reduction in the pericardial effusion. A cardiac MRI was performed (Figure 1) revealing a mildly dilated left ventricle, with moderate systolic dysfunction (LVEF: 36%), a hyperkinetic basal third, akinetic middle and apical third, and no signs of late enhancement, effectively ruling out myocarditis and integrating a diagnosis of takotsubo cardiomyopathy. Due to the clinical and laboratory improvement, the decision was made to discharge the patient with close clinical follow-up and cardiopulmonary rehabilitation.

Outcome and follow-up

Close monitoring was maintained with weekly medical consultations, resulting in the resolution of symptoms and the normalization of laboratory abnormalities, including NT-proBNP. A follow-up echocardiogram was performed four weeks later, which reported a reversal of all previous changes. The diuretic was discontinued, and the patient continued with low-dose angiotensin-converting enzyme (ACE) inhibitors and beta-blockers for maintenance.

## Discussion

We report on a case of biventricular takotsubo cardiomyopathy (BiTCM) secondary to multiple systemic inflammatory syndrome (MIS-A) in a 59-year-old female with recent second-dose vaccination for SARS-CoV-2 and elevated anti-SPIKE antibody titers without laboratory evidence of COVID-19. The diagnosis was made upon recognition of a temporal relationship between the administration of the BNT162b2 vaccine and the beginning of the first symptom, the nonspecific systemic inflammatory response syndrome, and the unusual biventricular systolic dysfunction associated with apical segmental akinesia revealed in the second echocardiographic evaluation.

This presentation meets the Centers for Disease Control and Prevention (CDC) case definition for MIS-A, which requires clinical and laboratory criteria occurring within the first three days of hospitalization (Table 2) [6]. This entity, developed in 2021 in response to the increasing number of cases of systemic inflammatory disease with end-organ involvement related to the SARS-CoV-2 pandemic, represents 0.2% of all COVID-19 complications [7]. In a systematic review by Kunal et al., 79 cases of MIS-A were analyzed, showing heterogeneous clinical features, with a predominance for cardiovascular involvement (81%), and a relatively good prognosis (5.1% mortality) [8]. When compared to other inflammatory complications associated with COVID-19, patients appeared to be older, with a higher risk for respiratory failure, cardiovascular and renal complications, invasive procedures (intubation and blood transfusion), and death [7]. The evolution is torpid, with patients showing a quick deterioration that results in ICU admission in 58.1%-100% of cases, usually due to cardiogenic shock [9]. This calls for quick identification and prompt initiation of supportive treatment, which typically involves ventilatory support, inotropes, fluid resuscitation, anticoagulation, and immunosuppression. Early corticosteroid therapy has been the mainstay therapy, with varied formulations being used [9,10].

**Table 2:** CDC case definition for multisystem inflammatory syndrome 2023 †Severe cardiac illness includes myocarditis, pericarditis, coronary artery dilatation/aneurysm, or new-onset right or left ventricular dysfunction (LVEF: <50%), second/third degree A-V block, or ventricular tachycardia. (Note: Cardiac arrest alone does not meet this criterion.) ‡New-onset neurological signs and symptoms include encephalopathy in a patient without prior cognitive impairment, seizures, meningeal signs, or peripheral neuropathy (including Guillain-Barré syndrome). CDC: Centers for Disease Control and Prevention, LVEF: left ventricular ejection fraction, SARS-CoV-2: severe acute respiratory syndrome coronavirus 2, IL-6: interleukin-6, RT-PCR: reverse transcription polymerase chain reaction Reference: Centers for Disease Control and Prevention. Multisystem inflammatory syndrome in adults (MIS-A) case definition information for healthcare providers. Centers for Disease Control and Prevention. January 3, 2023. Accessed June 1, 2023. https://www.cdc.gov/mis/mis-a/hcp.html.

Category	Description
A patient aged ≥21 years hospitalized for ≥24 hours, or with an illness resulting in death, who meets the following clinical and laboratory criteria.	
Clinical criteria	Subjective fever or documented fever (≥38.0°C) for ≥24 hours prior to hospitalization or within the first three days of hospitalization* and at least three of the following clinical criteria occurring prior to hospitalization or within the first three days of hospitalization*. At least one must be a primary clinical criterion. Primary clinical criteria: severe cardiac illness^†^, rash, and non-purulent conjunctivitis. Secondary clinical criteria: new-onset neurological signs and symptoms^‡^, shock or hypotension not attributable to medical therapy, and abdominal pain, vomiting, or diarrhea.
Secondary clinical criteria	The presence of laboratory evidence of inflammation and SARS-CoV-2 infection. Elevated levels of at least two of the following: C-reactive protein, ferritin, IL-6, erythrocyte sedimentation rate, and procalcitonin, and a positive SARS-CoV-2 test for current or recent infection by RT-PCR, serology, or antigen detection.

At the time of the second cardiac ultrasound, which revealed acute ventricular dysfunction, we had a strong suspicion of myocarditis, as Rosner et al. [11] recently reported a series of cases warning about messenger RNA (mRNA) vaccines potentially causing myocarditis as a rare side effect. Treatment for heart failure management was implemented. Due to the worsening clinical condition, we were unable to perform cardiac magnetic resonance imaging at that time. However, upon clinical improvement, a subsequent evaluation did not find evidence of myocarditis but rather identified biventricular takotsubo cardiomyopathy.

The reported case met the diagnostic criteria for MIS-A due to a biventricular systolic dysfunction, highly suggestive of takotsubo cardiomyopathy (TCM). This is a rare entity, with an estimated incidence of 0.02% of all hospitalizations and 2% of all acute coronary syndrome presentations [12]. Although heterogeneous, TCM diagnosis can be reached using the modified 2008 Mayo Clinic Criteria or the 2019 International Expert Consensus Document on Takotsubo Syndrome (Table 3) [13,14]. It has been proposed that TCM is associated with catecholamine toxicity, as well as myocardial ischemia and autonomic nervous system dysfunction [15].

**Table 3:** Takotsubo cardiomyopathy diagnostic criteria: comparison between the 2008 Modified Mayo Clinic Criteria and the 2019 International Expert Consensus Document on Takotsubo Syndrome †There are rare exceptions to these criteria, such as those patients in whom the regional wall motion abnormality is limited to a single coronary territory. ‡Wall motion abnormalities may remain for a prolonged period of time, or documentation of recovery may not be possible. §It is possible that a patient with obstructive coronary atherosclerosis may also develop ABS. However, this is very rare in our experience and in the published literature, perhaps because such cases are misdiagnosed as acute coronary syndrome. ¶Cardiac magnetic resonance imaging is recommended to exclude infectious myocarditis and diagnosis confirmation of takotsubo syndrome. ABS: apical ballooning syndrome, TTS: takotsubo syndrome, ECG: electrocardiogram References: Prasad A, Lerman A, Rihal CS. Apical ballooning syndrome (Tako-Tsubo or stress cardiomyopathy): a mimic of acute myocardial infarction. Am Heart J. 2008;155(3):408-417. doi: 10.1016/j.ahj.2007.11.008. Ghadri JR, Wittstein IS, Prasad A, et al. International Expert Consensus Document on Takotsubo Syndrome (Part I): Clinical Characteristics, Diagnostic Criteria, and Pathophysiology. Eur Heart J. 2018;39(22):2032-2046. doi:10.1093/eurheartj/ehy076.

Criteria	2008 Modified Mayo Clinic Criteria	2019 International Expert Consensus Document
1	Transient hypokinesis, akinesis, or dyskinesis of the left ventricular mid-segments with or without apical involvement; the regional wall motion abnormalities extend beyond a single epicardial vascular distribution; a stressful trigger is often, but not always present^†^.	Patients show transient^‡^ left ventricular dysfunction (hypokinesia, akinesia, or dyskinesia) presenting as apical ballooning or midventricular, basal, or focal wall motion abnormalities. Right ventricular involvement can be present. Besides these regional wall motion patterns, transitions between all types can exist. The regional wall motion abnormality usually extends beyond a single epicardial vascular distribution; however, rare cases can exist where the regional wall motion abnormality is present in the subtended myocardial territory of a single coronary artery (focal TTS).
2	Absence of obstructive coronary disease or angiographic evidence of acute plaque rupture^§^.	An emotional, physical, or combined trigger can precede the takotsubo syndrome event, but this is not obligatory
3	New electrocardiographic abnormalities (either ST-segment elevation and/or T-wave inversion) or modest elevation in cardiac troponin.	Neurological disorders (e.g., subarachnoid hemorrhage, stroke/transient ischemic attack, or seizures) and pheochromocytoma may serve as triggers for takotsubo syndrome.
4	Absence of pheochromocytoma and myocarditis	New ECG abnormalities are present (ST-segment elevation, ST-segment depression, T-wave inversion, and QTc prolongation); however, rare cases exist without any ECG changes.
5		Levels of cardiac biomarkers (troponin and creatine kinase) are moderately elevated in most cases; significant elevation of brain natriuretic peptide is common.
6		Levels of cardiac biomarkers (troponin and creatine kinase) are moderately elevated in most cases; significant elevation of brain natriuretic peptide is common.
7		Patients have no evidence of infectious myocarditis^‡^.
8		Postmenopausal women are predominantly affected.

Another important and highly relevant theory in this clinical case is the dysregulated corticosteroid hormonal balance, which can result in a maladaptive catecholaminergic response in cardiac tissue, leading to myocardial dysfunction and TCM. Both excessive and deficient levels of corticosteroids have been implicated in the development of TCM.

Recent literature supports this theory, including a case report by Batta et al. [16], which presented a unique case of autoimmune polyendocrine syndrome II manifesting as TCM. The authors discussed the pathophysiology behind this presentation and highlighted the role of dysregulated corticosteroid hormonal balance in the development of TCM. This case further supports the notion that imbalances in corticosteroid levels can contribute to the pathogenesis of TCM.

TCM and MIS-A are distinct clinical entities that require different treatment approaches. In the case of TCM, the mainstay of treatment is supportive care. The goal is to stabilize the patient's hemodynamics and manage symptoms. Medications such as beta-blockers and ACE inhibitors are commonly used to reduce cardiac workload and improve ventricular function. Diuretics may be prescribed to address fluid overload, and anticoagulation therapy may be considered to prevent thromboembolic events. Close monitoring of cardiac function through echocardiograms is essential to assess the recovery of ventricular dysfunction. Psychological support and cardiac rehabilitation may also play a role in the patient's overall management [17].

Certainly, coronary angiography is a useful resource in the diagnosis of TCM. However, the patient declined the invasive procedure of coronary angiography due to distress caused by hospitalization, the series of non-invasive studies performed, and the limited information about the multisystem inflammatory syndrome at the time of diagnosis. We support the diagnosis of takotsubo cardiomyopathy in this case based on the recent publication of the 2019 International Expert Consensus Document for TCM (Table 3). These criteria require radiological evidence of transient left ventricular dysfunction leading to apical ballooning or wall motion abnormalities, which was objectively demonstrated via magnetic resonance imaging in this patient.

Nevertheless, there were no clear indications of myocardial injury evident in either the electrocardiogram or significant troponin elevations. The contrast-enhanced cardiac magnetic resonance imaging revealed systolic dysfunction, with a hyperkinetic apical third and an akinetic basal third. No significant coronary obstruction was observed during the administration of the contrast.

On the other hand, adult multisystem inflammatory syndrome (MIS-A) is a condition characterized by systemic inflammation and multiorgan involvement. Treatment of MIS-A requires a multidisciplinary approach. The primary focus is on controlling the inflammatory response and providing supportive care for affected organ systems. Intravenous corticosteroids are commonly administered to dampen the immune response. Other immunomodulatory therapies, such as intravenous immunoglobulins (IVIGs), may also be considered. Additionally, targeted therapies aimed at specific organ involvement, such as cardiac medications for myocardial dysfunction or respiratory support for respiratory compromise, are employed as needed [18]. Close monitoring of clinical parameters and laboratory markers of inflammation is crucial during the treatment of MIS-A. The response to therapy should be assessed regularly, and adjustments may be made based on the patient's clinical course. Long-term follow-up is important to evaluate the resolution of symptoms and address any potential complications that may arise.

An important differential diagnosis in the presented patient is the relationship of osimertinib with cardiotoxicity. The published literature reports on varied clinical presentations, including acute-onset heart failure, atrial fibrillation, QT prolongation, acute myocardial infarction, and pericardial effusion [19]. Although the patient presented with a prolonged corrected QT, we attribute the finding to the patient's underlying hypocalcemia related to vomiting and zoledronic acid for the risk of metastatic fractures. In addition, the occurrence of drug-induced cardiac toxicity is less likely than the presented diagnosis in the context of a patient with strong evidence for an underlying hyperinflammatory state.

We theorize that the unusual BiTCM seen in this patient is a direct result of the interplay between the complex, pro-inflammatory state conferred by her past medical history of stage IV lung cancer, angioedema, Epstein-Barr infection, and idiopathic CD4+ lymphopenia, and the recent administration of the mRNA BNT162b2 SARS-CoV-2 vaccine, resulting in an intense, unregulated inflammatory state. Although severe, inflammatory side effects of the COVID-19 vaccine are extremely rare, and cases of allergic reactions, myocarditis, and other severe adverse effects have been reported [20,21].

Overall, the management of TCM and MIS-A involves a tailored approach that considers the individual patient's presentation, underlying conditions, and response to treatment. Collaboration among various medical specialties is essential to provide comprehensive care and optimize patient outcomes.

## Conclusions

This case represents the first reported instance of biventricular takotsubo cardiomyopathy (TCM) as the primary clinical criteria of a multisystemic inflammatory syndrome linked to the SARS-CoV-2 mRNA vaccine. Early recognition and treatment are vital for a positive outcome, emphasizing the need for prompt management and close clinical monitoring.

While this presentation could potentially be associated with the vaccine, it is important to note that it occurred in a patient with multiple comorbidities, antineoplastic medications, and advanced malignancy with immunosuppression. Thus, other factors may have contributed to the overall presentation. The case report does not establish a causal relationship with vaccine use and should be acknowledged accordingly.

This case highlights the importance of a comprehensive and multidisciplinary evaluation involving internal medicine, cardiology, and immunology specialists to effectively address and manage potential vaccine-related complications. Furthermore, it emphasizes the ongoing need for research to better understand the underlying mechanisms of these adverse reactions, enhancing vaccine safety and optimizing medical care for similar cases in the future.

## Figures and Tables

**Figure 2:**
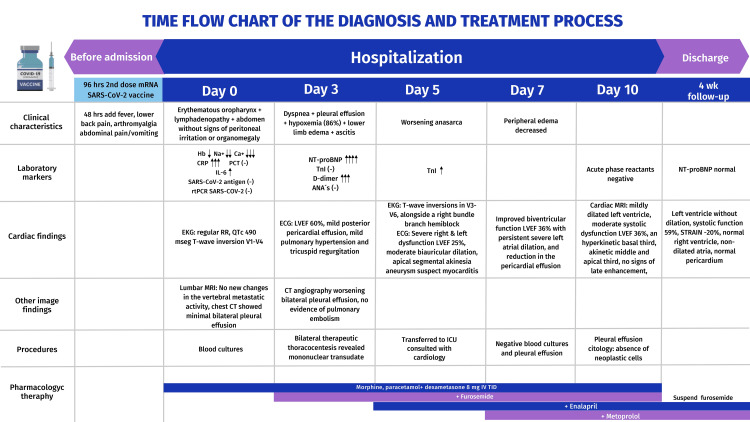
Time flowchart of the diagnosis and treatment process Hb: hemoglobin, Na+: sodium, Ca+: calcium, CRP: C-reactive protein, PCT: procalcitonin, IL-6: interleukin-6, TnI: troponin I, ANA: antinuclear antibody, EKG: electrocardiogram, RR: regular rhythm, QTc: corrected QT interval, ECG: echocardiogram, LVEF: left ventricular ejection fraction, MRI: magnetic resonance imaging, CT: computed tomography, ICU: intensive care unit, IV: intravenous, TID: three times a day

## Appendices

The time flowchart of the diagnosis and treatment process of the patient is shown in Figure 2.
